# Taxonomy and Phylogenetic Relationships of *Clavulinopsis* (Clavariaceae, Agaricales): Description of Six New Species and One Newly Recorded Species from China

**DOI:** 10.3390/jof9060656

**Published:** 2023-06-12

**Authors:** Jun Yan, Jing Wen, Gui-Wu Li, Shao-Wu Wu, Ping Zhang

**Affiliations:** 1 College of Life Sciences, Hunan Normal University, Changsha 410006, China; alexis830@163.com (J.Y.); 18163799060@163.com (J.W.); liguiwu201909@163.com (G.-W.L.); 2 Bureau of Forestry, Tongdao Dong Autonomous County, Huaihua 418500, China

**Keywords:** clavarioid fungi, Clavariaceae, phylogenetic analysis, morphology, taxonomy

## Abstract

Specimens of C*lavulinopsis* (Clavariaceae, Agaricales) collected in China were studied using morphological and molecular methods. Six species—*C*. *aspersa*, *C*. *bicolor*, *C*. *bispora*, *C*. *erubescens*, *C*. *incarnata*, and *C*. *tropicalis*—are described as new to science, and *C*. *trigonospora* is a newly recorded species in China. Phylogenetic analysis was conducted based on a combined dataset of internal transcribed spacer and nuclear ribosomal RNA large subunit sequences. The phylogenetic reconstruction revealed that the six new species each formed an independent lineage, and the samples of *C*. *trigonospora* from China were nested with accessions of *C*. *trigonospora* collected from Italy. The morphology of the seven Chinese species is described in detail, and is illustrated with line drawings and photographs. A key to the known *Clavulinopsis* species in China is provided.

## 1. Introduction

The genus *Clavulinopsis* Overeem comprises 34 species of coral fungi distributed worldwide [1] and classified in the Clavariaceae (Agaricales, Basidiomycota). Among the 165 taxon records for the genus currently listed in the Index Fungorum database (http://www.indexfungorum.org; accessed on 15 April 2023), approximately 70 species names are legitimately published. However, the majority of taxa lack molecular evidence and some even lack adequate micromorphological data. For example, *Clavulinopsis coliformis* (Boud.) Corner was transferred to *Clavulinopsis* by E.J.H. Corner in 1950, but E.J.H. Corner considered that *C*. *coliformis* might only be an unusual state of a well-known species, such as *C*. *corniculata* (Schaeff.) Corner or *C*. *fusiformis* (Sowerby) Corner [2]. Similarly, the taxonomic placement of other *Clavulinopsis* species, such as *C*. *pusilla* (Coker) Corner, *C*. *subflava* (Britzelm.) Corner, *C*. *sulphurascens* (Schwein.) Corner, and *C*. *tenella* (Boud.) Corner, is dubious [2]. In addition, the relationships among *Clavaria* Vaill. ex L., *Clavulinopsis*, and *Ramariopsis* (Donk) Corner, which are classified in the same family, have historically been controversial. Considering macromorphological evidence, the basidiome of the species placed in *Clavaria* and *Clavulinopsis* are simple or branched, clavarioid, and variously colored, whereas those of *Ramariopsis* species are rarely simple [2,3]. Based on micromorphological data, R.H. Petersen noted that *Clavaria* and *Clavulinopsis* are not separable at the genus rank because clamp connections are present on the basidia and hyphae in *Clavulinopsis*, but this is the sole character used to distinguish the genus from *Clavaria*. With regard to *Ramariopsis*, R.H. Petersen suggested that the genus must be segregated from *Clavaria* and *Clavulinopsis* on account of its branched fruit bodies and echinulate spores [4].

With the increase in taxonomic means, the boundaries between these genera are gradually being resolved. D.N. Pegler and T.W.K. Young [5] used scanning electron microscopy to examine the basidiospore structure in *Clavulinopsis* and *Ramariopsis* and observed that, among the species examined, most *Clavulinopsis* species were smooth-spored except for *Clavulinopsis helvola* (Pers.) Corner, whereas no species of *Ramariopsis* was smooth-spored. Furthermore, the mode of formation of basidiospore ornamentation was entirely different between *Ramariopsis* species and *Clavulinopsis* species. Birkebak et al. [6] conducted a molecular phylogenetic analysis to clarify the phylogenetic resolution of *Clavaria*, *Clavulinopsis*, and *Ramariopsis*. Thus, the status of *Clavulinopsis* as a distinct genus has been clarified, and knowledge of the similarities and differences in *Clavulinopsis* from related genera is much improved.

Reports on the taxonomic diversity in *Clavulinopsis* have been extremely limited in the past decade, with only four new species formally described (*C*. *aurantiaca* Araujo-Neta, G.A. Silva & Gibertoni, *C. dimorphica* A.N.M. Furtado & M.A. Neves, *C*. *imperata* A.N.M. Furtado & M.A. Neves, and *C*. *trigonospora* Franchi & M. Marchetti) [7,8,9]. Moreover, the sequence data generated for the *Clavulinopsis* species remain limited in quantity and coverage. In the present study, six new species and one newly recorded species of *Clavulinopsis* in China are described based on morphological examination and molecular phylogenetic analysis. Thirty-four newly generated sequences (seventeen ITS and seventeen nrLSU) for these seven *Clavulinopsis* species have been deposited in GenBank.

## 2. Materials and Methods

### 2.1. Specimens

Seventeen specimens of *Clavulinopsis* were collected from Hainan, Hunan, Jilin, Shaanxi, Sichuan, and Yunnan provinces in China from 2014 to 2022. The fresh specimens were dried using heat or indicating silica gel. The dried vouchers were deposited in the Mycological Herbarium of Hunan Normal University (MHHNU), Changsha, China. Detailed information on the 17 specimens and other samples used in the phylogenetic analysis are provided in Table 1, including GenBank accession numbers and locality information.

### 2.2. DNA Extraction, Amplification, and Sequencing

Total genomic DNA was extracted from the dried samples using the Ezup Column Fungi Genomic DNA Purification Kit (Sangon Biotech, Shanghai, China) in accordance with the manufacturer’s instructions. The primer pair ITS4/ITS5 [10] was used to amplify the internal transcribed spacer (ITS) region, and the universal primers LR0R, LR3, and LR5 [11,12] were used to amplify the nuclear ribosomal large subunit (nrLSU) region. Each PCR amplification was performed with an Eppendorf Mastercycler thermal cycler (Eppendorf Inc., Hamburg, Germany) in a 25 μL reaction mixture, which contained 1× PCR buffer, 1.5 mM MgCl_2_, 0.2 mM dNTPs, 0.4 μM of each primer, 1.25 U Taq polymerase (Sangon Biotech, Shanghai, China), and 1 μL DNA template. The thermal cycling was performed as follows: initial denaturation at 94 °C for 4 min, then 34 cycles of 94 °C for 40 s, annealing at 55 °C for 40 s, extension at 72 °C for 1 min, and a final extension at 72 °C for 8 min [13]. All amplified PCR products were electrophoresed on 1% agarose gel, and the purified PCR products were sequenced by Sangon Biotech (Shanghai, China). The specified primers were also used for sequencing reactions. All sequences newly generated in this study were submitted to GenBank.

### 2.3. Morphological Studies

The macromorphological characters of species were based on field notes and habitat photographs. Color codes used in descriptions follow Kornerup and Wanscher [14], and color terms are from Ridgway [15]. The micromorphological characters were observed with a light microscope. Tissue sections of dried materials were first rehydrated with 5% KOH solution or distilled water, and then stained with 1% Congo Red when necessary. The notation [*n*/*m*/*p*] means that *n* basidiospores were measured from *m* basidiomes of *p* specimens. The basidiospore dimensions are described using the notation (**a**–)**b**–**c**(–**d**). The range **b**–**c** contains a minimum of 90% of the measured values, and **a** and **d** are extreme values, provided in parentheses. The *Q* value is the length/width ratio of each basidiospore, and the **Q** value is the average *Q* ± standard deviation.

### 2.4. Alignment and Phylogenetic Analyses

Sequence data for the legitimate species of the genus *Clavulinopsis* and two *Mucronella* species were downloaded from GenBank. The downloaded and newly generated sequences constituted the dataset for the present analysis. The ITS and nrLSU sequences were, respectively, aligned using the default settings for gap openings and gap extension penalties with MAFFT v7.471 [16], and then manually adjusted where necessary in BIOEDIT v7.2.5 [17]. The concatenated ITS–nrLSU data matrix containing 79 sequences (39 ITS and 40 nrLSU) was assembled with SEQUENCEMATRIX 1.7.8 [18]. A maximum likelihood (ML) analysis was conducted using RAXML v8.0.20 [19] with 1000 bootstrap replicates and the GTR + Gamma evolutionary model. Bayesian inference (BI) was performed with MRBAYES v3.2.7 [20] and run for 1,000,000 generations with the GTR + I + G optimal evolutionary model selected with MRMODELTEST v2.4 [21] for each partition, using four Markov Chain Monte Carlo (MCMC) chains to calculate posterior probabilities. The tree files were merged and edited with FIGTREE v1.4.2 [22].

**Table 1:** Voucher information and GenBank accession numbers of taxa used in this study.

Identification	Specimen No.	GenBank No. (ITS)	GenBank No. (28S)	Location	References
*Clavulinopsis amoena*	PBM3381	—	HQ877702	Australia: Tasmania	Birkebak et al. [6]; Hyde et al. [8]
*C. appalachiensis*	S.D. Russell iNaturalist # 91596164	OM809324	—	USA: Indiana	Unpublished
*C. appalachiensis*	TENN074980	MT196965	—	USA: Tennessee	Unpublished
***C. aspersa***	**MHHNU10153**	**OQ703777**	**OQ703794**	**China: Hunan**	**Present study**
***C. aspersa***	**MHHNU10342**	**OQ703778**	**OQ703795**	**China: Hunan**	**Present study**
***C. aspersa***	**MHHNU11103**	**OQ703779**	**OQ703796**	**China: Hunan**	**Present study**
*C. aurantiaca*	URM <BRA>: 84212	—	KX227749	Brazil: Pernambuco	Hyde et al. [8]
*C. aurantiaca*	URM <BRA>: 84216	KC348464	NG058946	Brazil: Pernambuco	Hyde et al. [8]
*C.* aff. *aurantiocinnabarina*	JMB08171004	—	HQ877704	USA: Tennessee	Birkebak et al. [6]; Hyde et al. [8]
*C.* aff. *aurantiocinnabarina*	JMB08240901	—	HQ877703	USA: Tennessee	Birkebak et al. [6]; Hyde et al. [8]
***C. bicolor***	**MHHNU10381**	**OQ703780**	**OQ703797**	**China: Hainan**	**Present study**
***C. bispora***	**MHHNU11181**	**OQ703781**	**OQ703798**	**China: Jilin**	**Present study**
***C. bispora***	**MHHNU11188**	**OQ703782**	**OQ703799**	**China: Jilin**	**Present study**
*C. corallinorosacea*	PBM3380	KP257144	HQ877707	Australia: Tasmania	Birkebak et al. [6,23]; Hyde et al. [8]
*C. corniculata*	TENN064106	KP257145	HQ877713	USA: Tennessee	Birkebak et al. [6,23]; Hyde et al. [8]
*C. corniculata* f. *bispora*	AMB 18573	MT055953	—	Italy	Franchi and M. Marchetti [9]
***C. erubescens***	**MHHNU8040**	**OQ703783**	**OQ703800**	**China: Hunan**	**Present study**
***C. erubescens***	**MHHNU10290**	**OQ703784**	**OQ703801**	**China: Shaanxi**	**Present study**
*C. fusiformis*	**HKAS122627**	ON794403	—	China	Wang et al. [24]
*C. fusiformis*	PBM 2804	—	EF535273	USA: Massachusetts	Birkebak et al. [6]; Hyde et al. [8]
*C. fusiformis*	TENN064110	—	HQ877717	USA: Tennessee	Birkebak et al. [6]; Hyde et al. [8]
*C. fusiformis*	2728	—	KM248914	**—**	Unpublished
*C. gracillima*	MO 215748	KY706170	—	Canada: Windsor	Hay et al. [25]
*C. gracillima*	TENN065662	—	HQ877708	USA: Tennessee	Unpublished
*C. helvola*	EL 111/04	EU118617	EU118617	Sweden	Birkebak et al. [6]; Hyde et al. [8]
*C. helvola*	Lueck6	KP965770	KP965788	Germany: Lueckendorf	Karich et al. [26]
***C. incarnata***	**MHHNU9314**	**OQ703785**	**OQ703802**	**China: Hunan**	**Present study**
***C. incarnata***	**MHHNU9813**	**OQ703786**	**OQ703803**	**China: Yunnan**	**Present study**
***C. incarnata***	**MHHNU11330**	**OQ703787**	**OQ703804**	**China: Yunnan**	**Present study**
***C. incarnata***	**MHHNU11331**	**OQ703788**	**OQ703805**	**China: Yunnan**	**Present study**
*C. laeticolor*	EL 8/00	EU118618	EU118618	Finland	Birkebak et al. [6]; Hyde et al. [8]
*C. luteoalba*	BRACR16669	—	JQ415959	Denmark: Copenhagen	Hyde et al. [8]
*C. luteoalba*	BSI13 147a	OP538704	—	Switzerland	Unpublished
*C. miyabeana*	ZP-2118	MK427059	—	China: Hunan	Chen and Zhang, P. [27]
*C.* sp.	MCCNNU 00948	MT587808	MT587810	China	Unpublished
*C.* sp.	MCCNNU 00952	MT587809	MT587811	China	Unpublished
*C. sulcata*	PBM3379	—	HQ877709	Australia: Tasmania	Birkebak et al. [6]; Hyde et al. [8]
*C. sulcata*	PDD78241	—	DQ284904	New Zealand	Dentinger, B.T.M. and McLaughlin, D.J. [28]
*C. trigonospora*	AMB: 18557	NR176720	NG088120	Italy: Capezzano Pianore	Franchi and M. Marchetti [9]
*C. trigonospora*	AMB: 18587	—	MT055968	Italy: Capezzano Pianore	Franchi and M. Marchetti [9]
***C. trigonospora***	**MHHNU9186**	**OQ703789**	**OQ703806**	**China: Sichuan**	**Present study**
***C. trigonospora***	**MHHNU9200**	**OQ703790**	**OQ703807**	**China: Sichuan**	**Present study**
***C. trigonospora***	**MHHNU10198**	**OQ703791**	**OQ703808**	**China: Gansu**	**Present study**
***C. tropicalis***	**MHHNU10721**	**OQ703792**	**OQ703809**	**China: Hainan**	**Present study**
***C. tropicalis***	**MHHNU10722**	**OQ703793**	**OQ703810**	**China: Hainan**	**Present study**
*C. umbrinella*	HFRG_EJ191214_3_FRDBI 17588114	OQ133539	OQ133591	United Kingdom: Hampshire	Unpublished
*Ramariopsis laeticolor*	CR12764	**—**	GU299509	Slovakia	Unpublished
*R. laeticolor*	UBC F23885	KJ146701	**—**	Canada: British Columbia Area	Unpublished
*Mucronella flava*	IO.16.84	MT232354	MT232307	Sweden	Olariaga et al. [29]; Yan et al. [30]
*Mucronella* sp.	PDD95742	HQ533013	**—**	New Zealand	Yan et al. [30,31]

Note: Newly generated sequences are shown in bold.

## 3. Results

### 3.1. Taxonomy

**Figure 1:**
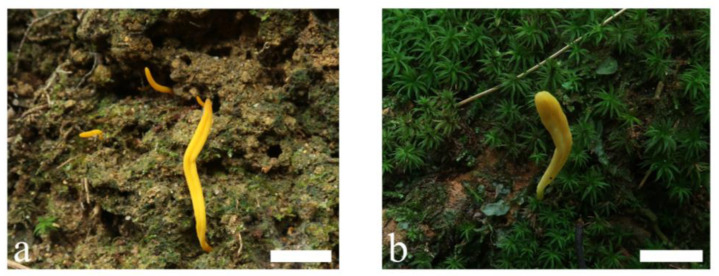
Basidiomata of *Clavulinopsis aspersa* (**a**) MHHNU10342; (**b**) MHHNU11103. Scale bars = 2 cm.

**Figure 2:**
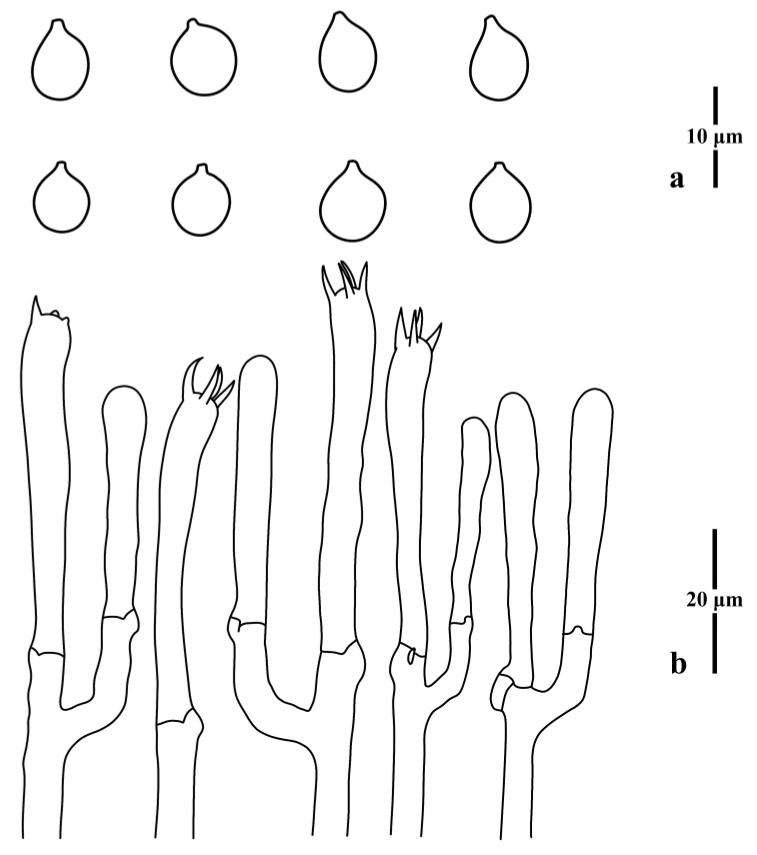
Microscopic features of *Clavulinopsis aspersa* (MHHNU10342). (**a**) Basidiospores; (**b**) basidia.

***Clavulinopsis aspersa*** P. Zhang & Jun Yan, sp. nov.: Figure 1 and Figure 2.

MycoBank: 848958

Diagnosis: Characterized by its solitary or scattered habit, yellowish to yellow basidiomata, and smooth, thin-walled, broadly ellipsoid to ellipsoid basidiospores.

Etymology: *aspersa* (Lat.) refers to the scattered growth habit of this species.

Type: China. Hunan Province: Rucheng County, Jiulongjiang National Forest Park, 25°26′44.01″ N, 113°47′09.58″ E, alt. 500 m, in broadleaved forest, 22 June 2020, Ping Zhang (MHHNU10342, holotype).

Description: Basidiomata fragile, simple, 15–50 mm tall, 1–4 mm wide, solitary or scattered to gregarious, rarely caespitose-connate at the base. Fertile part claviform or subcylindric to fusiform, sometimes slightly curved or flexuous, occasionally longitudinal depressions or grooves when old, yellowish [1A3–4, Matius Yellow, Picric Yellow, Pale Greenish Yellow] to yellow [2A7–8, Apricot Yellow, Light Cadmium] with age. Apex rounded, concolorous, becoming yellow [2A7–8, Light Cadmium] to dark yellow [4A7–8, Aniline Yellow]. Sterile part narrow or indistinct, concolorous or slightly paler, sometimes semitransparent, without tomentum or mycelial patch at the base. Context fragile, hymenium concolorous. Taste, odor, and macrochemical reactions were not recorded.

Basidiospores [100/4/3] (6.0) 6.5–8.0 (8.5) × 5.0–6.0 μm [*Q* = 1.17–1.40 (1.50), **Q** = 1.26 ± 0.08], thin-walled, hyaline, smooth, inamyloid, broadly ellipsoid to ellipsoid with a distinct apiculus. Basidia (36) 42–56 × 6.0–8.0 μm, thin-walled, hyaline, clavate to subcylindrical, with a clamp connection, four tapered sterigmata, 3.5–7.0 μm long. Basidioles, incrustations, or crystals absent. Subhymenium clearly delimited from the context, composed of densely interwoven hyphae. Hyphae of the context cylindrical to inflated, thin-walled, hyaline, parallel, without secondary septa, with clamp connections. Hyphae near subhymenium 1.5–5.0 μm wide; hyphae distant from subhymenium 8.0–10.0 μm wide.

Habitat and distribution: Solitary or scattered to gregarious, on soil rich in humus, in broadleaved forest, known only in subtropical areas of China; June to July.

Additional specimens examined: China. Hunan Province: Guzhang County, Laoyapo, 28°41′13.69″ N, 110°05′20.16″ E, alt. 953 m, in broadleaved forest, 22 July 2019, Ping Zhang (MHHNU10153); Tongdao County, Fengshuwan Forest Park, 26°09′45.66″ N, 109°46′31.52″ E, alt. 400 m, in broadleaved forest, 6 July 2022, Ping Zhang (MHHNU11103).

Comments: In the genus *Clavulinopsis*, approximately 10 species are similar to *C*. *aspersa* in the basidioma color. Among these species, *C*. *dimorphica* and *C*. *fleischeriana* (Henn.) Corner, with many-branched basidiomata, are immediately distinguishable [2,7]. *Clavulinopsis helvola* and *C*. *trigonospora* are particularly distinctive in *Clavulinopsis* because the former species has a spiny spore and the latter has a subtriangular spore [2,9]. According to published data, the *Q* of spores of *C*. *antillarum* (Pat.) Courtec. are 1.0–1.18 [32], and the average *Q* of spores of *C*. *imperata* are 1.08 [7], which indicates that *C*. *aspersa* has significantly narrower spores (*Q* = 1.17–1.40). The irregularly thick-walled hyphae of *C*. *imperata* are unique within *Clavulinopsis* [7]. Based on spore dimensions, *C*. *luteoalba* (Rea) Corner (5.0–8.0 × 2.5–4.5 μm or 5.2–8.0 × 2.8–4.4 μm) can be distinguished from *C*. *aspersa* [2,9]. Compared with *C*. *aspersa*, *C*. *amoena* (Zoll. & Moritzi) Corner, *C*. *fusiformis*, and *C*. *laeticolor* (Berk. & M.A. Curtis) R.H. Petersen often grow more densely and are very variable in form, size, and color. Moreover, *C*. *amoena* is found in tropical areas from sea level to 1300 m and has subglobose spores (4.0–7.0 × 4.0–6.5 μm); *C. fusiformis* has a taller basidioma (5–14 cm) and slightly thick-walled spores; and *C. laeticolor* has slightly thick-walled hyphae and spores [2].

**Figure 3:**
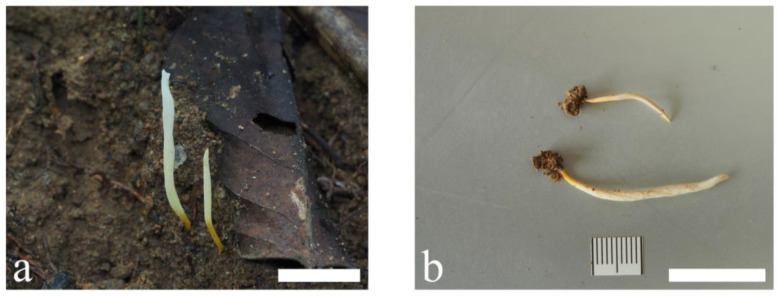
Basidiomata of *Clavulinopsis bicolor* (**a**,**b**) MHHNU10381. Scale bars = 2 cm.

**Figure 4:**
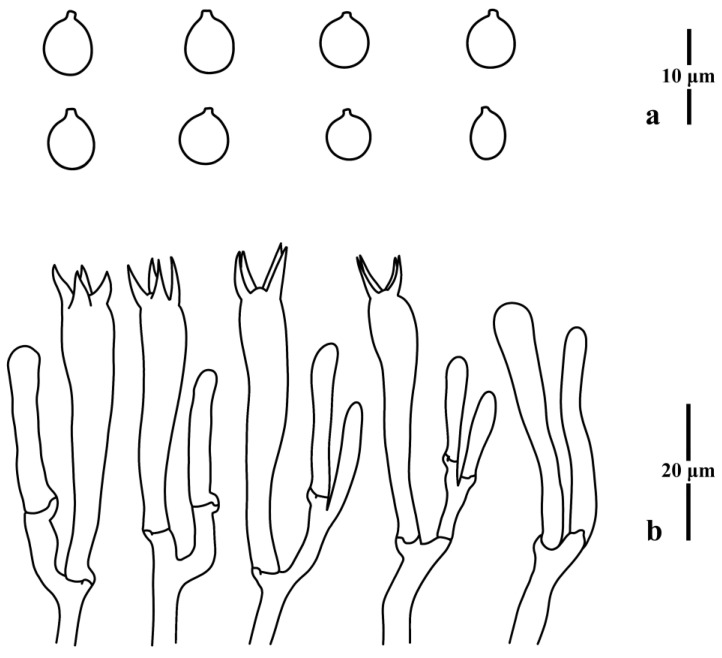
Microscopic features of *Clavulinopsis bicolor* (MHHNU10381). (**a**) Basidiospores; (**b**) basidia.

***Clavulinopsis bicolor*** P. Zhang & Jun Yan, sp. nov.: Figure 3 and Figure 4.

MycoBank: 848959

Diagnosis: Characterized by a simple, bicolored basidiomata and smooth, thin-walled, globose or subglobose basidiospores.

Etymology: *bicolor* (Lat.) refers to the two different colors of the basidiomata, namely, yellow to brown at the base and white or creamy white to pale green-white above.

Type: China. Hainan Province: Baoting County, Qixianling, 18°42′04.60″ N, 109°41′57.61″ E, alt. 350 m, in tropical broadleaved forest, 26 June 2020, Sainan Li (MHHNU10381, holotype).

Description: Basidiomata fragile, simple, 20–40 mm tall, 1–2 mm wide, scattered to gregarious; the base is divided. Fertile part subcylindric to fusiform, sometimes slightly twisted and with longitudinal depressions, white or creamy white to pale green-white [1A1, 26A2, White, Pale Glaucous-Green, Pale Blue-Green]. When dried, the color changes to pale yellow-white [4A2, Sea-Form Yellow]. Apex obtuse–acute, concolorous, becoming yellow [4A2–3, Martius Yellow]. Sterile part narrow, very distinct, 5–7 mm tall, yellow to brown [2A4–7, Yellowish Citrine, Dark Olive-Buff], without tomentum or mycelial patch at the base. Context fragile, hymenium concolorous. Taste, odor, and macrochemical reactions were not recorded.

Basidiospores [60/2/1] (4.5) 4.7–5.5 (5.7) × (4.0) 4.4–5.0 μm [*Q* = 1.04–1.15 (1.25), **Q** = 1.09 ± 0.05], thin-walled, hyaline, smooth, inamyloid, globose or subglobose, with a distinct apiculus. Basidia (32) 37–46 × (5.0) 5.0–7.0 μm, thin-walled, hyaline, clavate to subcylindrical, with a clamp connection, four tapered sterigmata, 4.0–8.5 μm long. Basidioles, incrustations, or crystals absent. Subhymenium clearly delimited from the context, composed of densely interwoven hyphae. Hyphae of the context cylindrical to inflated, thin-walled, hyaline, parallel, without secondary septa, with clamp connections. Hyphae near subhymenium 1.0–6.0 μm wide; hyphae distant from subhymenium 8.0–10.0 μm wide.

Habitat and distribution: Scattered to gregarious, on soil rich in humus, in tropical broadleaved forest, known only in a tropical area of China; June.

Comments: *C*. *bicolor* is most difficult to distinguish from *C*. *appalachiensis* (Coker) Corner and *C*. *rufipes* (G.F. Atk.) Corner because all have a brown stem and light-colored fertile parts. Based on morphological records, *C*. *appalachiensis* not only shows a certain difference in color from *C*. *bicolor*, but also has a larger basidioma (3.0–9.0 × 0.15–0.5 cm), a longer stem (1–4 cm), and broader basidia (7.0–8.5 μm) than *C*. *bicolor*. Compared with *C*. *appalachiensis*, *C*. *rufipes* is more similar to *C*. *bicolor* in macromorphology except that it occasionally has sparingly branched basidiomata. However, *C*. *rufipes* have pip-shaped to ovoid spores, and shorter basidia (23–30 μm) than *C*. *bicolor* [2].

**Figure 5:**
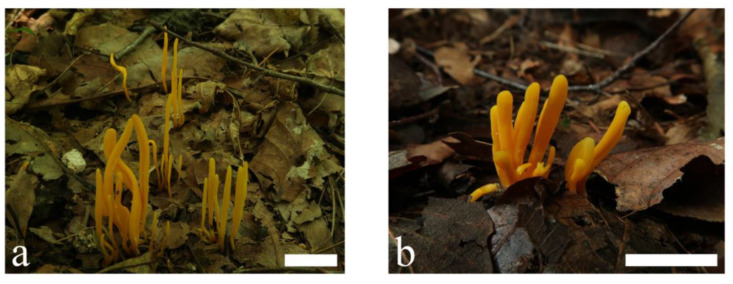
Basidiomata of *Clavulinopsis bispora* (**a**) MHHNU11188; (**b**) MHHNU11181. Scale bars = 2 cm.

**Figure 6:**
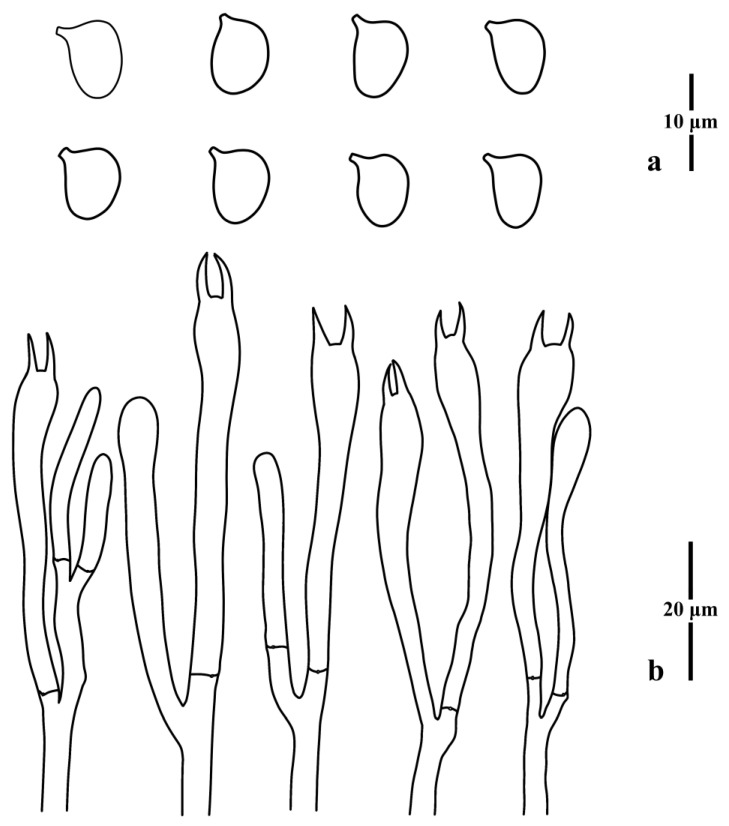
Microscopic features of *Clavulinopsis bispora* (MHHNU11188). (**a**) Basidiospores; (**b**) basidia.

***Clavulinopsis bispora*** P. Zhang & Jun Yan, sp. nov.: Figure 5 and Figure 6.

MycoBank: 848960

Diagnosis: Differs from other taxa in the genus by having very small clamp connections and two-spored basidia.

Etymology: *bispora* (Lat.) refers to the two-spored basidia.

Type: China. Jilin Province: Tonghua, Baijifeng National Forest Park, 41°33′57.68″ N, 126°04′47.00″ E, alt. 680 m, in broadleaved forest, 6 Auguest 2022, Ping Zhang (MHHNU11188, holotype).

Description: Basidiomata fragile, simple, 30–50 mm tall, 1–3 mm wide, gregarious or caespitose. Fertile part subcylindric to claviform, sometimes conspicuously twisted and with a distinct longitudinal depression, yellowish [1A5–6, Lemon Yellow, Empire Yellow] to orange-yellow [1A7–8, Aniline Yellow, Ochraceous Orange] with age. Apex rounded, concolorous, becoming slightly darker when mature. Sterile part narrow, indistinct, concolorous or slightly differing in color from the upper part, occasionally semitranslucent, without tomentum or mycelial patch at the base. Context fragile, hymenium concolorous or slightly paler. Taste, odor, and macrochemical reactions were not recorded.

Basidiospores [100/4/2] 6.0–8.0 (8.5) × (4.5) 5.0–6.0 μm [*Q* = (1.08) 1.17–1.60 (1.70), **Q** = 1.37 ± 0.16], thin-walled, hyaline, smooth, inamyloid, most ellipsoid to broadly ellipsoid, several long ellipsoid or subglobose with a distinct apiculus. Basidia (42) 46–55 × 5.0–7.0 μm, thin-walled, hyaline, clavate to subcylindrical, with a very small clamp connection, two tapered sterigmata, 5.0–8.0 μm long. Basidioles, incrustations, or crystals absent. Subhymenium clearly delimited from the context, composed of densely interwoven hyphae. Hyphae of the context cylindrical to inflated, thin-walled, hyaline, parallel, without secondary septa, with clamp connections. Hyphae near subhymenium 2.0–5.0 μm wide; hyphae distant from subhymenium ~12 μm wide.

Habitat and distribution: Gregarious or caespitose, on soil rich in humus, in broadleaved forests, known only in northeast China; August.

Additional specimen examined: China. Jilin Province: Ji’an County, Wunvfeng National Forest Park, 41°16′19.37″ N, 126°08′14.46″ E, alt. 700 m, in broadleaved forest, 5 Auguest 2022, Ping Zhang (MHHNU11181).

Comments: The characteristics of small clamp connections and two-spored basidia are extremely rare in *Clavulinopsis*, and long sterigmata is also not a typical feature in the genus. According to previous records, *C*. *calocera* (G.W. Martin) Corner, *C*. *inflatissima* Corner, *C*. *lingula* Corner, and *C*. *sibutiana* (Har. & Pat.) Corner have small clamps [2,3]. However, *C*. *calocera* was transferred to the genus *Lepidostroma* Mägd. & S. Winkl. [33]. *C*. *inflatissima* and *C*. *lingula* have large spores (9.0–11.5 × 8.0–9.0 μm and 10.0–12.0 × 6.0 μm, respectively) and four-spored basidia [3], and *C*. *sibutiana* is close to *C*. *lingula* and has been considered a variant of *C*. *lingula* [3]; thus, these species are easily distinguished from *C*. *bispora*. With regard to other taxa with two-spored basidia, *C*. *corniculata* f. *bispora* may produce a profusely branched basidioma, the hyphae and basidia lack clamps [2,9], and *C*. *luticola* is more specialized on account of its short basidia (8–12 μm long) and flask-shaped cystidia [2].

**Figure 7:**
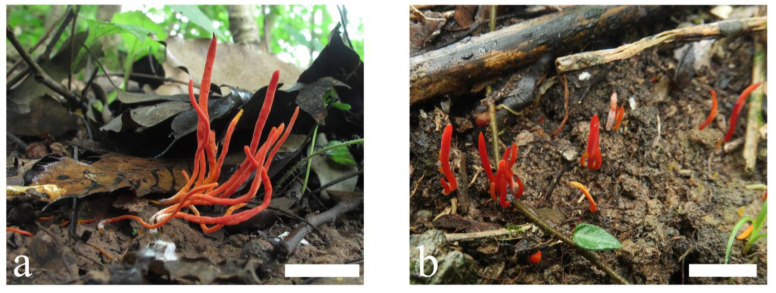
Basidiomata of *Clavulinopsis erubescens* (**a**) MHHNU8040; (**b**) MHHNU10290. Scale bars = 2 cm.

**Figure 8:**
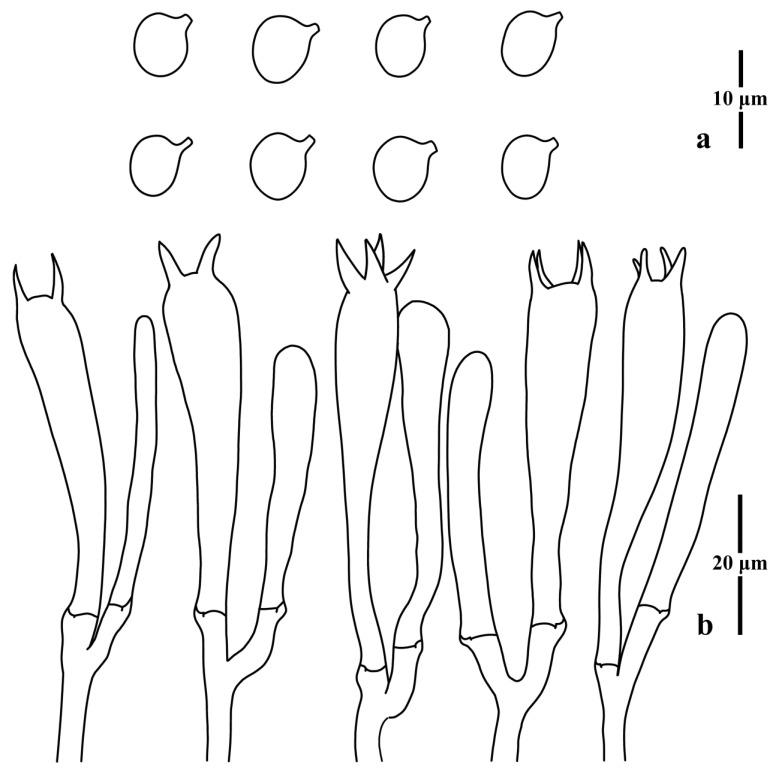
Microscopic features of *Clavulinopsis erubescens* (MHHNU8040). (**a**) Basidiospores; (**b**) basidia.

***Clavulinopsis erubescens*** P. Zhang & Jun Yan, sp. nov.: Figure 7 and Figure 8.

MycoBank: 848961

Diagnosis: Distinguished from other taxa in the genus by its simple red basidiomata, ellipsoid to broadly ellipsoid basidiospores, and two-spored or four-spored basidia.

Etymology: *erubescens* (Lat.) alludes to the basidiomata becoming red with age.

Type: China. Hunan Province: Yuanling County, Fenghuangshan Forest Park, 28°27′10.41″ N, 110°25′27.32″ E, alt. 150 m, in broadleaved forest, 25 June 2014, Ping Zhang (MHHNU8040, holotype).

Description: Basidiomata fragile, simple, 10–60 mm tall, 1–4 mm wide, gregarious to caespitose. Fertile part subcylindric to fusiform, occasionally slightly curved or flexuous and with a distinct longitudinal depression, red-orange [7A7–8, 7B7–8, Coral Red, Peach Red, Grenadine Red] to red [10A7–8, 10B7–8, Scarlet Red, Rose Doree, Corinthian Red]. Apex rounded or obtuse–acute when mature, concolorous or slightly paler, becoming darker with age. Sterile part narrow, indistinct, concolorous or paler than the upper part, occasionally curved, without tomentum or mycelial patch at the base. Context fragile, hymenium concolorous. Taste, odor, and macrochemical reactions were not recorded.

Basidiospores [100/4/2] (5.5) 5.8–7.0 × (4.3) 4.5–5.2 μm [*Q* = 1.20–1.40 (1.44), **Q** = 1.29 ± 0.07], thin-walled, hyaline, smooth, inamyloid, ellipsoid to broadly ellipsoid with a distinct apiculus. Basidia (37) 40–54 × 6–8 μm, thin-walled, hyaline, clavate to subcylindrical, clamped, two or four tapered sterigmata, 4.0–6.0 μm long. Basidioles, incrustations, or crystals absent. Subhymenium clearly delimited from the context, composed of densely interwoven hyphae. Hyphae of the context cylindrical to inflated, thin-walled, hyaline, parallel, without secondary septa, with clamp connections. Hyphae near subhymenium 1.0–5.0 μm wide; hyphae distant from subhymenium ~8 μm wide.

Habitat and distribution: Gregarious to caespitose, on soil rich in humus, in broadleaved forests, only known in Hunan and Shaanxi provinces, China; June to October.

Additional specimen examined: China. Shaanxi Province: Lueyang County, Baishigou Village, 33°22′28.74″ N, 106°12′05.14″ E, alt. 900 m, in broadleaved forest, 4 October 2019, Ping Zhang (MHHNU10290).

Comments: Within the genus *Clavulinopsis*, *C*. *corallinorosacea*, *C*. *depokensis* (Overeem) Corner, *C*. *miyabeana* (S. Ito) S. Ito, and *C*. *sulcata* are similar to *C*. *erubescens* in having a simple red basidioma. However, compared with *C*. *erubescens*, *C*. *corallinorosacea* has a taller, occasionally branched basidioma and narrower basidiospores; *C*. *depokensis* has four-spored basidia and is specialized among *Clavulinopsis* species in occurring on dead leaves and twigs in woods rather than on soil. Additionally, *C*. *sulcata* has a variably colored basidioma and globose or subglobose basidiospores [2]. Consistent with the morphological differences, the present phylogenetic analysis confirmed that *C*. *erubescens* has only a distant affinity with, and is placed in a separate lineage to, *C*. *corallinorosacea*, *C*. *miyabeana*, and *C*. *sulcata*.

**Figure 9:**
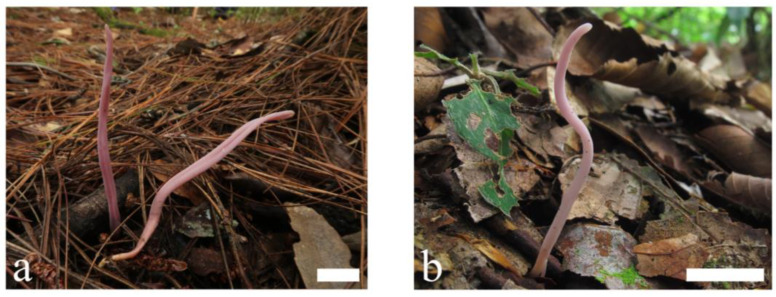
Basidiomata of *Clavulinopsis incarnata* (**a**) MHHNU11330; (**b**) MHHNU9314. Scale bars = 2 cm.

**Figure 10:**
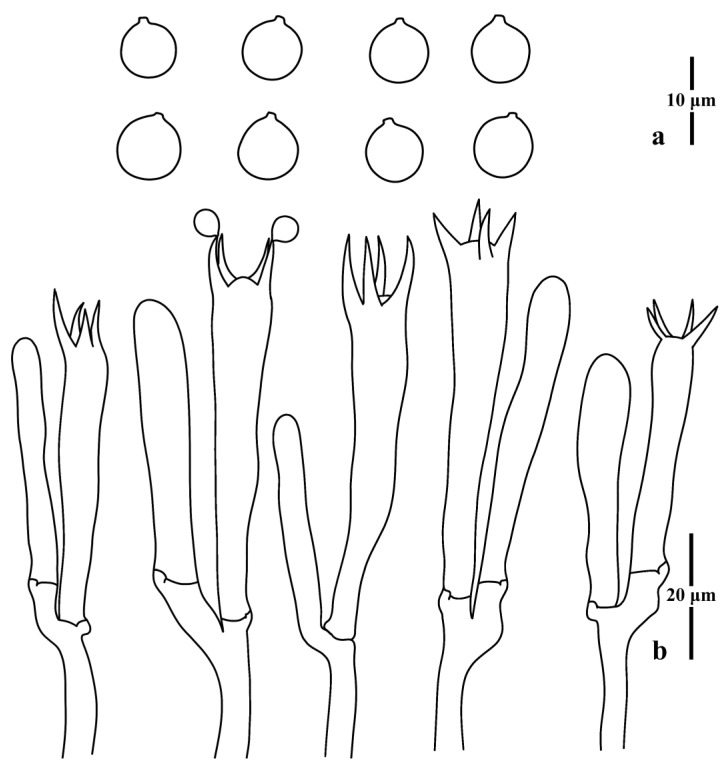
Microscopic features of *Clavulinopsis incarnata* (MHHNU11330). (**a**) Basidiospores; (**b**) basidia.

***Clavulinopsis incarnata*** P. Zhang & Jun Yan, sp. nov.: Figure 9 and Figure 10.

MycoBank: 848962

Diagnosis: Differs from other taxa in the genus in having a pinkish basidioma, four-spored basidia, globose to subglobose basidiospores, and very long sterigmata.

Etymology: *incarnata* (Lat.) refers to the pinkish basidiomata of this species.

Type: China. Yunnan Province: Binchuan County, Jizu Moutain, 25°57′28.20″ N, 100°23′17.37″ E, alt. 2300 m, in mixed coniferous–broadleaved forest, 29 Auguest 2022, Ping Zhang (MHHNU11330, holotype).

Description: Basidiomata fragile, simple, 30–120 mm tall, 1–7 mm wide, solitary or scattered to gregarious. Fertile part subcylindric to fusiform, sometimes conspicuously twisted and with a distinct longitudinal depression, pinkish [11A2–3, 12A4–5, Hermosa Pink, Eosine Pink Thulite Pink]. Apex rounded or obtuse–acute, obviously paler than the lower part, becoming pale yellow or brown with age. Sterile part narrow, indistinct, concolorous or subtly different in color, without tomentum or mycelial patch at the base. Context fragile, hymenium concolorous or slightly paler. Taste, odor, and macrochemical reactions were not recorded.

Basidiospores [100/4/4] 6.0–7.5 (8.0) × 6.0–7.0 (8.0) μm [*Q* = 1.00–1.09 (1.15), **Q** = 1.05 ± 0.04], thin-walled, hyaline, smooth, inamyloid, globose to subglobose with a distinct apiculus. Basidia (36) 43–61 × 8.0–11.0 μm, thin-walled, hyaline, clavate to subcylindrical, clamped, four tapered sterigmata, 6.5–13.0 μm long. Basidioles, incrustations, or crystals absent. Subhymenium clearly delimited from the context, composed of densely interwoven hyphae. Hyphae of the context cylindrical to inflated, thin-walled, hyaline, parallel, without secondary septa, with clamp connections. Hyphae near subhymenium 1.5–5.0 μm wide; hyphae distant from subhymenium 8.0–12.5 μm wide.

Habitat and distribution: Solitary or scattered to gregarious, on soil rich in humus, in broadleaved forest or mixed coniferous–broadleaved forest, known only in subtropical areas of China; August to September.

Additional specimens examined: China. Hunan Province: Sangzhi County, Badagong Moutain, 29°40′49.64″ N, 109°48′24.81″ E, alt. 1300 m, in broadleaved forest, 14 September 2017, Ping Zhang (MHHNU9314); Yunnan Province: Malipo County, 23°21′41.98″ N, 105°09′44.17″ E, alt. 1580 m, in broadleaved forest, 3 Auguest 2019, Ping Zhang (MHHNU9813); Binchuan County, Jizu Moutain, 25°57′28.20″ N, 100°23′17.37″ E, alt. 2300 m, in mixed coniferous–broadleaved forest, 29 Auguest 2022, Ping Zhang (MHHNU11331).

Comments: Based on the simple or sparsely branched, pinkish basidiomata, this species may be mistaken for *Clavaria incarnata* Weinm., *Clavaria rosea* Fr., *Clavulina amethystinoides* (Peck) Corner, and *Clavulina purpurascens* P. Zhang. However, as it possesses the generic characters of four-spored basidia without postpartal septation and clamped hyphae, it can be determined that *Clavulinopsis incarnata* is assignable to *Clavulinopsis* [2,3,13]. The genus *Clavulinopsis* includes several species with a pinkish basidioma. Compared with *C*. *incarnata*, *C*. *alcicornis* (Zoll. & Moritzi) Corner, *C*. *carneola* Corner, *C*. *lignicola* (R.H. Petersen) Corner, *C*. *lingula*, and *C*. *moricolor* Corner all have a shorter basidioma. In addition, *C*. *alcicornis* mainly has three-spored basidia and *C*. *carneola* has four-spored or six-spored basidia; *C*. *lignicola* generally has a bifurcate basidioma and smaller spores (2.8–3.2 × 2.1–2.3 μm) similar to those of *C*. *moricolor* (3.3–4.2 × 2.5–3.0 μm); and *C*. *lingula* has unique clamp connections [2,3]. *C*. *corallinorosacea* (Cleland) Corner and *C*. *sulcata* Overeem are more similar to *C*. *incarnata* in basidiomata size, but the basidiomata of these two species often grows more densely. In addition, the former species has a narrower, fusiform or amygdaliform basidiospore, and the basidiomata of the latter species is thinly white and villous at the whitish base [2,3].

**Figure 12:**
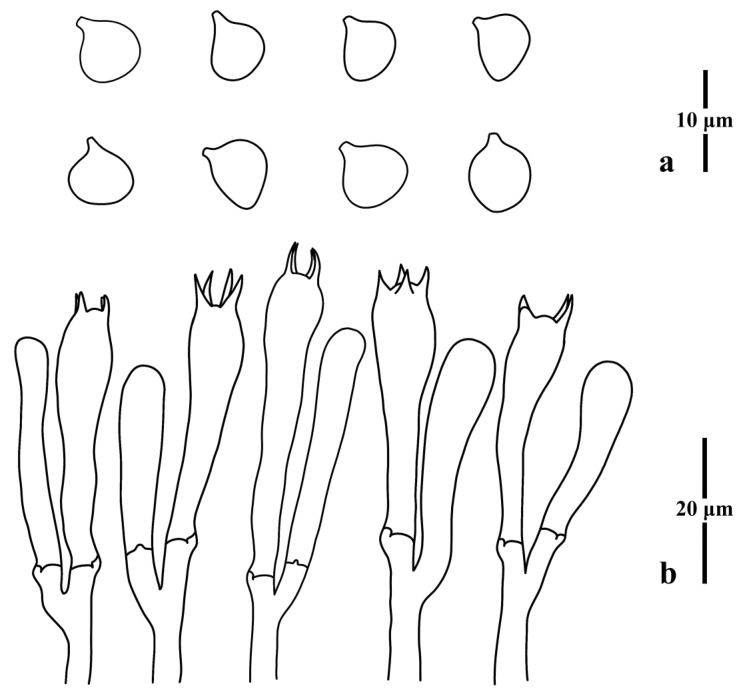
Microscopic features of *Clavulinopsis trigonospora* (MHHNU9200). (**a**) Basidiospores; (**b**) basidia.

***Clavulinopsis trigonospora*** Franchi & M. Marchetti, Index Fungorum 457:1, 2020: Figure 11 and Figure 12.

Diagnosis: Distinguished from other taxa in the genus by its smooth, thin-walled, subtriangular, quadrilateral to subglobose basidiospores.

Description: Basidiomata fragile, simple, 6–90 mm tall, 1–4 mm wide, solitary or scattered to gregarious. Fertile part subcylindric, clavate to fusiform, sometimes curved or flexuous and with a longitudinal depression, yellowish to yellow [2A6–8, 30A5–6, Empire Yellow, Lemon Yellow, Clear Dull Green Yellow]. Apex rounded, concolorous, becoming darker than the lower part with age. Sterile part indistinct, concolorous or semitransparent, without tomentum or mycelial patch at the base. Context fragile, hymenium concolorous. Taste, odor, and macrochemical reactions were not recorded.

Basidiospores [100/4/3] 5.8–7.5 (8.0) × 4.5–6.5 μm [*Q* = 1.21–1.51, **Q** = 1.29 ± 0.08], thin-walled, hyaline, smooth, inamyloid, subtriangular to ellipsoid with a distinct apiculus. Basidia (30) 32–43 × 6–9 (10) μm, thin-walled, hyaline, clavate to subcylindrical, clamped, four tapered sterigmata, 5.0–7.0 μm long. Basidioles, incrustations, or crystals absent. Subhymenium clearly delimited from the context, composed of densely interwoven hyphae. Hyphae of the context cylindrical to inflated, thin-walled, hyaline, parallel, without secondary septa, with clamp connections. Hyphae near subhymenium 2.0–4.0 μm wide; hyphae distant from subhymenium 6.0–10.0 μm wide.

Habitat and distribution: Solitary or scattered to gregarious, on soil rich in humus, in broadleaved forest or mixed coniferous–broadleaved forest, known in Italy and China; August to November.

Specimens examined: China. Sichuan Province: Kangding County, Jiefang Second Village, 30°28′47.48″ N, 102°15′46.38″ E, alt. 2860 m, in mixed coniferous–broadleaved forest, 20 Auguest 2017, Ping Zhang (MHHNU9186, MHHNU9200); Gansu Province: Yuzhong County, Xinglongshan National Nature Reserve, 35°47′55.36″ N, 104°04′20.27″ E, alt. 2450 m, in broadleaved forest, 7 Auguest 2019, Ping Zhang (MHHNU10198).

Comments: Based on the records of Franchi and M. Marchetti [9], *C*. *trigonospora* has a simple, bright yellow to light yellow-orange, small basidioma (25–45 mm tall, 1–2 mm wide), and its most distinctive character is subtriangular spores. Compared with the type specimen, our specimens collected in China show a larger range in size, but all possess subtriangular spores. The distribution of this species in China was confirmed by phylogenetic analysis of molecular data, and thus the present account is the first record of this species outside of Italy.

**Figure 13:**
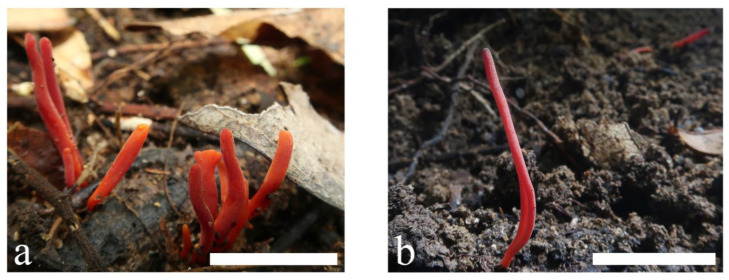
Basidiomata of *Clavulinopsis tropicalis* (**a**) MHHNU10721; (**b**) MHHNU10722. Scale bars = 2 cm.

**Figure 14:**
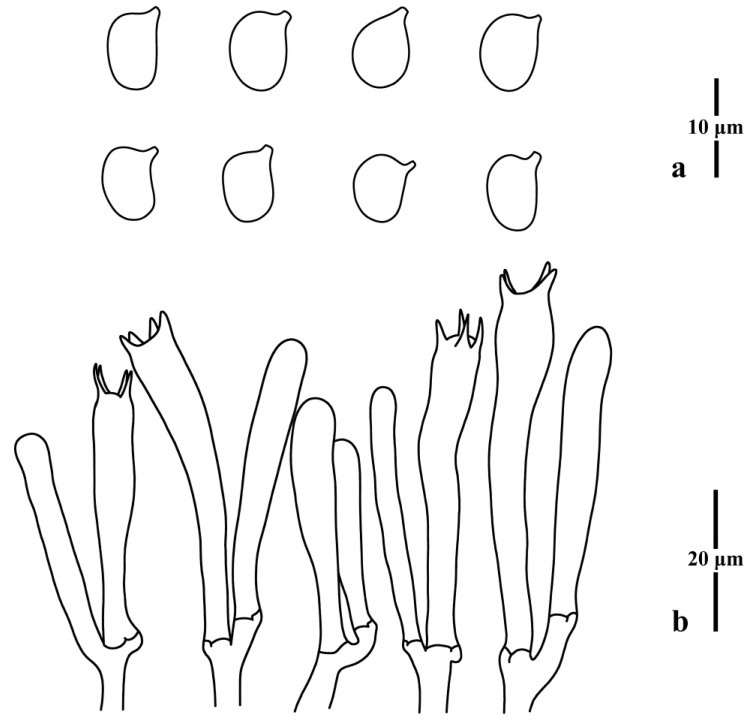
Microscopic features of *Clavulinopsis tropicalis* (MHHNU10722). (**a**) Basidiospores; (**b**) basidia.

***Clavulinopsis tropicalis*** P. Zhang & Jun Yan, sp. nov.: Figure 13 and Figure 14.

MycoBank: 848963

Diagnosis: The species has a red basidioma and ellipsoid to broadly ellipsoid basidiospores, and differs from *C*. *erubescens* in having only four-spored basidia and growing in the tropics.

Etymology: *tropicalis* (Lat.) refers to the climate in which the species was discovered.

Type: China. Hainan Province: Baoting County, Qixianling, 18°42′08.44″ N, 109°41′36.19″ E, alt. 300 m, in tropical broadleaved forest, 31 July 2021, Ping Zhang (MHHNU10722, holotype).

Description: Basidiomata fragile, simple, 15–35 mm tall, 1–3 mm wide, solitary or gregarious to caespitose. Fertile part subcylindric to fusiform, occasionally slightly curved or flexuous and with a distinct longitudinal depression when mature, red to dark red [10B7–8, 10C7–8, Acajou Red, Pompeian Red] or pinkish [11A5–7, Spinel Pink, Spinel Red, Corinthian Red]. Apex rounded, concolorous or slightly paler, becoming darker with age. Sterile part narrow, indistinct, concolorous or paler than the upper part, occasionally curved, without tomentum or mycelial patch at the base. Context fragile, hymenium concolorous. Taste, odor, and macrochemical reactions were not recorded. Basidiospores [100/4/2] 5.0–7.0 (8.5) × 4.0–5.5 μm [*Q* = 1.18–1.50, **Q** = 1.29 ± 0.12] thin-walled, hyaline, smooth, inamyloid, broadly ellipsoid to ellipsoid with a distinct apiculus. Basidia (35) 43–50 × 6–9 μm, thin-walled, hyaline, clavate to subcylindrical, clamped, four tapered sterigmata, 4.0–7.0 μm long. Basidioles, incrustations, or crystals absent. Subhymenium clearly delimited from the context, composed of densely interwoven hyphae. Hyphae of the context cylindrical to inflated, thin-walled, hyaline, parallel, without secondary septa, with clamp connections. Hyphae near subhymenium 1.5–3.5 μm wide; hyphae distant from subhymenium 8.0–10.5 μm wide.

Habitat and distribution: Solitary or gregarious to caespitose, on soil rich in humus, in tropical broadleaved forest, known only in tropical areas of China; July.

Additional specimen examined: China. Hainan Province: Baoting County, Qixianling, 18°42′08.44″ N, 109°41′36.19″ E, alt. 300 m, in tropical broadleaved forest, 31 July 2021, Ping Zhang (MHHNU10721).

Comments: Compared with *C*. *erubescens*, *C*. *tropicalis* is distributed in the tropics and only has four-spored basidia. Based on the present multi-loci phylogenetic analysis, although *C*. *erubescens* and *C*. *tropicalis* are placed on sister branches, there was a genetic distance of 0.0400 between the two taxa.

### 3.2. Molecular Phylogenetic Analysis

**Figure 15:**
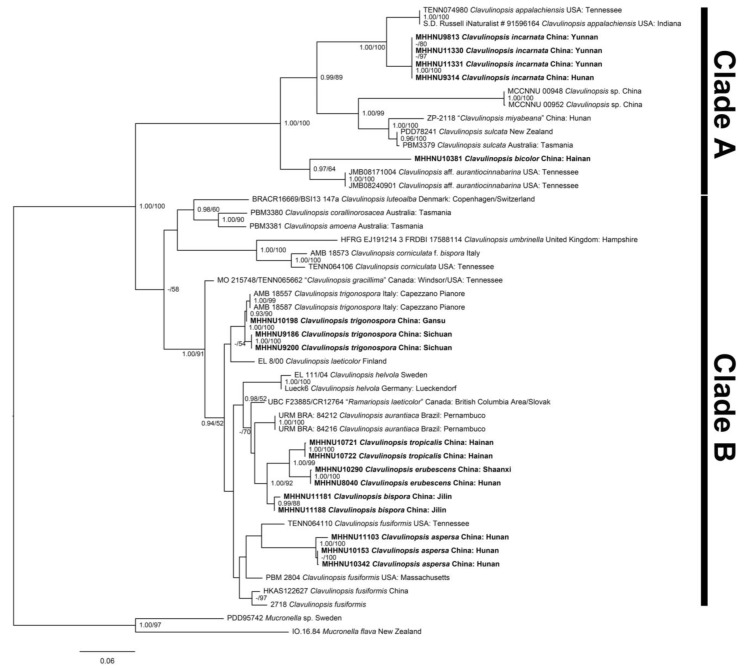
Phylogenetic relationships of *Clavulinopsis* species inferred from the combined dataset (ITS and nrLSU) using Bayesian posterior probabilities ≥ 0.90; ML Bootstrap values ≥ 50% are reported on the branches; the sign “-” means under the reported level. Six new species and one newly recorded species are shown in boldface text.

In total, 79 sequences (39 ITS and 40 nrLSU) from 50 samples (Table 1) were assembled into a multi-gene dataset for the molecular phylogenetic analysis, of which 34 sequences (17 ITS and 17 nrLSU) were newly generated in the present study. The combined ITS–nrLSU dataset consisted of 1612 sites and represented 23 species of *Clavulinopsis* and two species of *Mucronella* as the outgroup. The ML and BI (not shown) phylogenies (Figure 15) were extremely similar in topology and included two resolved clades (A and B) among the species of *Clavulinopsis*. The monophyly of clade A was strongly supported (PP = 1.00, BP = 100%) and included *C*. *appalachiensis* (PP = 1.00, BP = 100%), *C*. *incarnata* (PP = 1.00, BP = 100%), one unnamed *Clavulinopsis* species (PP = 1.00, BP = 100%), *C*. *miyabeana*, *C*. *sulcata* (PP = 0.96, BP = 100%), *C*. *bicolor*, and *C*. aff. *aurantiocinnabarina* (PP = 1.00, BP = 100%). Clade B (BP= 58%) comprised 16 species: *C*. *luteoalba*, *C*. *corallinorosacea*, *C*. *amoena*, *C*. *umbrinella*, *C*. *corniculata* (PP = 1.00, BP = 100%), *C*. *gracillima* (dubious name), *C*. *trigonospora* (PP = 1.00, BP = 100%), *C*. *laeticolor*, *C*. *helvola* (PP = 1.00, BP = 100%), *R*. *laeticolor* (dubious name), *C*. *aurantiaca* (PP = 1.00, BP = 100%), *C*. *tropicalis* (PP = 1.00, BP = 100%), *C*. *erubescens* (PP = 1.00, BP = 100%), *C*. *bispora* (PP = 1.00, BP = 94%), *C*. *aspersa* (PP = 1.00, BP = 100%), and *C*. *fusiformis*. The six new species each formed an independent lineage, and the newly collected samples of *C*. *trigonospora* from China were nested within *C*. *trigonospora* accessions collected from Italy.

## 4. Discussion

In this study, six *Clavulinopsis* species new to science and one *Clavulinopsis* species newly recorded in China were identified. The specimens of *Clavulinopsis trigonospora* from Sichuan and Gansu, as a newly recorded species in China, conformed with the type specimen from Italy in molecular phylogenetic placement and morphological characteristics, and represent the first discovery of this species outside of Italy. Morphology and molecular data verified that the other six species collected were new species. The six new *Clavulinopsis* species are herein formally named and described in detail, and are illustrated with line drawings and photographs.

In the process of conducting the phylogenetic analysis, we found that available sequences of *Clavulinopsis* species are still extremely limited in GenBank and many species are represented by only one or two accessions with sequences. After adding the newly generated sequences in this study, we obtained a more stable topology for *Clavulinopsis*, with two clades resolved, in comparison with the previous works by Birkebak et al. [6] and Hyde et al. [8]. However, the common synapomorphic traits for species within clade A and clade B currently remain unclear and, in any case, samples of additional taxa are needed for reconstruction of a taxonomically complete phylogeny.

Prior to the present study, no new *Clavulinopsis* species had been described in China. Most *Clavulinopsis* specimens previously collected in China were assigned to species originating in Europe, Oceania, or the Americas. Based on our field investigations and molecular analyses, it is apparent that the main difficulties hindering the classification of *Clavulinopsis* are the currently limited availability of molecular data and the paucity of reliable, taxonomically important morphological characters. The present study has provided additional molecular data, provided a key to the known *Clavulinopsis* species in China (Table A1) and enhanced knowledge of the biological diversity in *Clavulinopsis*. However, the diversity of the genus in China is much richer than expected and additional research is needed to further explore this diversity in the future.

## Figures and Tables

**Figure 11:**
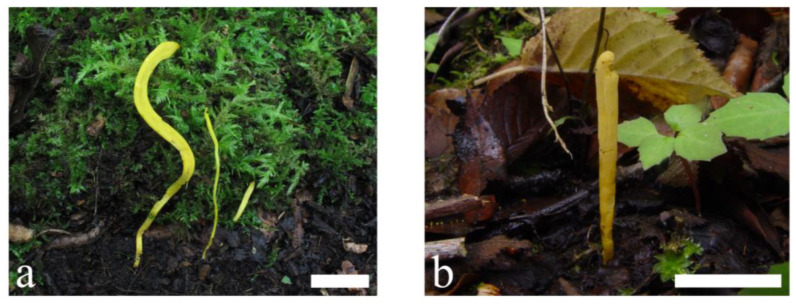
Basidiomata of *Clavulinopsis trigonospora* (**a**) MHHNU9186; (**b**) MHHNU9200. Scale bars = 2 cm.

## Data Availability

The sequence data generated in this study are deposited in NCBI GenBank.

## Appendix A

**Table A1:** Key to *Clavulinopsis* species in China.

1. Basidiomata branched	*C. corniculata*
1. Basidiomata unbranched	2
2. Basidiospores spinous	*C. helvola*
2. Basidiospores smooth	3
3. Basidiospores subtriangular to ellipsoid	***C. trigonospora***
3. Basidiospores globose, subglobose or ellipsoid	4
4. Basidia and hyphae with a very small clamp connection	***C. bispora***
4. Basidia and hyphae with a normal size clamp connection	5
5. Basidiomata bicolored	***C. bicolor***
5. Basidiomata monochromatic	6
6. Basidiomata red or pinkish	7
6. Basidiomata white, yellowish or orange	11
7. Basidiomata pinkish	***C. incarnata***
7. Basidiomata red	8
8. Basidia two or four tapered sterigmata	***C. erubescens***
8. Basidia invariably four tapered sterigmata	9
9. Basidiospores globose to subglobose	*C*. *sulcata*
9. Basidiospores ellipsoid, fusiform or amygdaliform	10
10. Basidiomata 15–35 mm tall, basidiospores broadly ellipsoid to ellipsoid	***C* *. tropicalis***
10. Basidiomata mostly 60–90 mm tall, basidiospores rather fusiform or amygdaliform	*C*. *corallinorosacea*
11. Basidiomata white or cream color	12
11. Basidiomata yellowish or orange	13
12. Basidiospores broadly pip-shaped, the wall slightly thickened	*C. brevipes*
12. Basidiospores subglobose, thin-walled	*C*. *spiralis*
13. Basidiomata orange, orange-red or salmon-orange	14
13. Basidiomata yellowish	15
14. Basidiomata 15–30 mm tall, basidiospores globose, rarely subglobose	*C. aurantiaca*
14. Basidiomata 15–70mm tall, basidiospores subglobose	*C. aurantiocinnabarina*
15. Distributed in the tropics	*C. amoena*
15. Distributed in the temperate and subtropics	16
16. Basidiospore hilar appendix not prominent (<1 μm)	*C. luteoalba*
16. Basidiospore hilar appendix very prominent (>1 μm)	17
17. Tramal hyphae somewhat thick-walled (up to 1 μm)	*C. laeticolor*
17. Tramal hyphae thin-walled	18
18. Basidiomata 50–140 mm tall, densely fasciculate	*C. fusiformis*
18. Basidiomata 15–50 mm tall, solitary or scattered to gregarious	***C* *. aspersa***
